# RU486 Metabolite Inhibits CCN1/Cyr61 Secretion by MDA-MB-231-Endothelial Adhesion

**DOI:** 10.3389/fphar.2019.01296

**Published:** 2019-11-20

**Authors:** Suhong Yu, Cuicui Yan, Wenjing Wu, Sudan He, Min Liu, Jian Liu, Xingtian Yang, Ji Ma, Yusheng Lu, Lee Jia

**Affiliations:** ^1^Cancer Metastasis Alert and Prevention Center, and Biopharmaceutical Photocatalysis of State Key Laboratory of Photocatalysis on Energy and Environment, College of Chemistry, Fujian Provincial Key Laboratory of Cancer Metastasis Chemoprevention and Chemotherapy, Fuzhou University, Fuzhou, China; ^2^Institute of Oceanography, Minjiang University, Fuzhou, China

**Keywords:** MDA-MB-231/HPMEC co-culture, metapristone, CYR61, integrin α_v_β_1_, metastasis chemoprevention

## Abstract

Successful adhesion of circulating tumor cells (CTCs) to microvascular endothelium of distant metastatic tissue is the key starting step of metastatic cascade that could be effectively chemoprevented as we demonstrated previously. Here, we hypothesize that the hetero-adhesion may produce secretory biomarkers that may be important for both premetastatic diagnosis and chemoprevention. We show that co-incubation of triple-negative breast cancer (TNBC) cell line MDA-MB-231 with human pulmonary microvascular endothelial monolayers (HPMEC) secretes Cyr61 (CCN1), primarily from MDA-MB-231. However, addition of metapristone (RU486 metabolite) to the co-incubation system inhibits Cyr61 secretion probably *via* the Cyr61/integrin αvβ1 signaling pathway without significant cytotoxicity on both MDA-MB-231 and HPMEC. Transfection of MDA-MB-231 with Cyr61-related recombinant plasmid or siRNA enhances or reduces Cyr61 expression, accordingly. The transfection significantly changes hetero-adhesion and migration of MDA-MB-231, and the changed bioactivities by overexpressed CYR61 could be antagonized by metapristone *in vitro*. Moreover, the circulating MDA-MB-231 develops lung metastasis in mice, which could be effectively prevented by oral metapristone without significant toxicity. The present study, for the first time, demonstrates that co-incubation of MDA-MB-231 with HPMEC secrets CYR61 probably *via* the CYR61/integrin α_v_β_1_ signaling pathway to promote adhesion-invasion of TNBC (early metastatic step). Metapristone, by interfering the adhesion-invasion process, prevents metastasis from happening.

## Introduction

Breast cancer is the most common cancer and the leading cause of related death among females worldwide (Akram et al., 2017; Barrios et al., 2018). Triple-negative breast cancer (TNBC) represents about 15% of all breast cancers and is largely refractory to current available therapies (Wein and Loi, 2017). Therefore, identifying biomarkers responsible for TNBC, and developing a novel cancer metastasis chemopreventive agent is critical for alternative breast cancer treatment approaches. Metastasis is a frequent occurrence in TNBC. The most important step in metastasis is the migration of cancer cells away from the primary tumor, and then adhesion to the endothelial cells, a process called tumor invasion (Valastyan and Weinberg, 2011). Studies indicate that the migration and invasion of pathogenesis of tumor cells depend on cross-communications between tumor cells and various endothelial cells residing in their microenvironment (Rodvold and Zanetti, 2016; Lambert et al., 2017). For example, several growth factors signaling pathways, secreted proteins or micro RNAs (miRNAs) and exosomes are functional mediators of tumor-endothelial interactions in metastasis (Miller and Grunewald, 2015; Yu et al., 2015a; Yu et al., 2015b; Zeng et al., 2018). However, the way invasive cancer cells diminish the endothelial barrier function still remains elusive.

Cysteine-rich protein 61 (CCN1/Cyr61), cysteine-rich, heparin-binding extracellular matrix-associated protein, is the first cloned member of cysteine-rich protein (CCN) family which includes connective tissue growth factor (CTGF, CCN2), nephroblastoma over-expressed protein (Nov, CCN3), Wnt-1-induced secreted protein 1 (WISP-1, CCN4), WISP-2 (CCN5) and WISP-3 (CCN6) (Lin et al., 2012; Jandova et al., 2012). As a secreted protein, Cyr61 connects with the extracellular matrix and the cell surface (Grzeszkiewicz et al., 2002) and is a communication media between cancer cells and the host which can reflect the changes arising due to cancer treatment (Bonin-Debs et al., 2004; Mbeunkui et al., 2006). The Cyr61 protein has been reported to mediate cell adhesion, stimulate chemostasis, augment growth factor-induced DNA synthesis, foster cell survival, and enhance angiogenesis (Hou et al., 2014). Overexpression of Cyr61 enhanced the growth and migration of glioma cells through activation of the ILK-mediated-catenin-TCF/Lef and the Akt signaling pathways (Wu et al., 2017). Silencing Cyr61 in invasive breast cancer cells caused a major loss of MMP-1 induction from stromal fibroblasts and inhibited the tumorigenicity of breast cancer cells (Nguyen et al., 2006). Researchers showed that Cyr61 can activate biochemical signal transduction through interacting with various integrins (Crockett et al., 2007; Su et al., 2010). While binding of integrin α_v_β_3_ triggered cell adhesion and apoptosis, binding of integrin α_6_β_1_ induced senescence, and binding of integrin α_v_β_5_ affected migration (Crockett et al., 2010; Lau, 2011). These reports indicated that the conformation of Cyr61 and integrins may play a vital role in metastasis.

Metapristone is the most predominant biological active metabolite of mifepristone (Heikinheimo et al., 1989; Teng et al., 2011), which has received considerable attention due to its anticancer activity in the recent years. Metapristone was developed as a novel cancer metastasis chemopreventive agent by us for its per-metastatic chemoprevention. In our previous studies, metapristone induced dose-dependent apoptosis, and interfered with adhesion of HT-29 cells to human umbilical vein endothelial cells (HUVECs) *in vitro* (Wang et al., 2014). Moreover, our studies demonstrated that metapristone inhibited TNBC cells migration and adhesion to endothelial cells through intervening the EMT-related signaling pathways (Yu et al., 2016). Inspired by our previous studies, we hypothesize that the anti-metastasis potential of metapristone is related with the bidirectional cross-talk between endothelial and tumor cells.

To test the hypothesis, we examined the effects of endothelial cells (HPMEC) on the aggressive phenotype of breast cancer cells (MDA-MB-231) using an *in vitro* co-culture system. We observed that the co-culture of HPMEC with MDA-MB-231 increased the expression of Cyr61 (CCN1), and the formation of Cyr61/integrin α_v_β_1_ complex. This highlights an important contact in cell communication between malignant breast epithelial cells and the endothelium. This study also supports our hypothesis and reveals a novel function for metapristone in the prevention of breast cancer metastasis by intervening Cyr61/integrin α_v_β_1_ signaling pathways. The study report is as follows.

## Materials and Methods

### Materials

Anti-Cyr61, Anti-ITGAV, Anti-ITGB1, and goat Anti-Rabbit lgG H&L were from Abcam. Human recombinant Cyr61 was obtained from GeneTex. SiRNA-Cyr61 and negative control siRNA were purchased from Sangon Biotech (Shanghai). Pierce Co-Immunoprecipitation (Co-IP) Kit (26149) was purchased from Thermo scientific. The recombinant plasmid of pcDNA3.1-Cyr61 was constructed by our lab.

### Cell Culture

MDA-MB-231 cells were obtained from American Type Culture Collection (ATCC, Manassas, VA), and were incubated with Leibovitz’s L-15 medium (Catalog No. 30-2008) containing 10% FBS, 100 U/ml penicillin and 100 µg/ml streptomycin at 37 °C in a free gas exchange with atmospheric air. MCF-7 cells were purchased from the national experimental cell resource sharing service platform (Beijing) and cultured in RPMI-1640 medium with 10% FBS, 100 U/ml penicillin, and 100 µg/ml streptomycin. Human pulmonary microvascular endothelial cells (HPMEC) were purchased from PromoCell, and cultured in ECM with 10% FBS, 100 U/ml penicillin, and 100 µg/ml streptomycin at 37 °C in 5% CO_2_ atmosphere.

### *In* *Vitro* Cytotoxicity Studies

The *in vitro* cytotoxicity was determined as what we described previously (Lau, 2011; Shi et al., 1993). MDA-MB-231 cells were trypsinized and seeded on 96-well plates at 8×10^3^ cells/well. After adhering of 24 h, doses of metapristone (0,10,25,50,75,100 µM) were added and incubated for another 12 and 24 h respectively. Then, 100 µl/well MTT (5 mg/ml) was added and incubated for 4 h in incubator. The MTT solution was aspirated and replaced with 100 µl/well dimethyl sulfoxide solution (DMSO). After shaking 10 to 30 min, the plates were measured at 570 nm using an infinite M200 Pro microplate reader (Tecan, Switzerland).

### Co-Culture Model and iTRAQ Analysis

Metastasis, a process that cancer cells invade surrounding tissues and migrate to distal organs including lung, liver, brain, bone, and lymph nodes, is a major cause of mortality in breast cancer patients (Torre et al., 2015), and adhering to the vascular endothelium is a key step when this process starts (Dotan et al., 2009). Thus, in this study, we used *co-culture* model to stimulate the tumor microenvironment *in vitro*. HPMEC cells were seeded in 75 cm flasks with complete ECM culture. After overnight adhesion, MDA-MB-231 cells (1:2) were plated into the same flasks with serum-free and metapristone (50 µM). Then the flasks were incubated another 24 h. Each group of serum-free ECM culture media was incubated for iTRAQ analysis. The procedures for iTRAQ and further analysis are described as these labs previously (Adav et al., 2010; Yu et al., 2015a; Yu et al., 2015b).

### Elisa Assay

Enzyme-linked immunosorbent assay (ELISA) was used to investigate the secretion levels of Cyr61 at different conditions. Concentrations of Cyr61 from each group were measured quantitatively using a sandwich ELISA as described (Adav et al., 2010). Briefly, 96-Well ELISA plates were coated with mouse anti-human Cyr61 (ab80112) and stored overnight at 4°C. After three washes in PBST, wells were blocked with 1% BSA in PBS-T for 2 h at room temperature. Next, the serum-free medium was added to duplicate wells and human recombination Cyr61 (GTX48189-PRO) protein was diluted into different concentrations with PBS as the standard. Simultaneously, an additional set of wells were coated with blank buffer to serve as a control. Then plates were incubated at 37°C for 2 h and followed by three washed in PBS-T, respectively. A rabbit anti-human Cyr61 mAb (Santa Cruz Biotechnology, sc-13100) was added and incubated at 37°C for 2 additional h. After washing, alkaline phosphatase-conjugated goat anti-rabbit IgG antibodies were added for 2 additional h, then 2 M sodium hydroxide solution were used for color development. Concentrations of Cyr61 were determined by detecting the absorbance at 405 nm using an infinite M200 Pro microquant plate reader (Tecan, Switzerland). Each test was repeated at least three times.

### Cell Morphology Assay

5×10^4^ HPMEC cells were cultured in a 35 mm cell culture dish (NEST, GBD-35-20) for 12 h and then co-cultured with 2.5×10^4^ MDA-MB-231 cells with or without metapristone (50 µM). This system was taken for a time-lapse photography by the Leica TCS SP8 confocal microscope.

### Plasmid Construction, siRNAs Synthesis, and Transient Transfection

The ORF of the human Cyr61 cDNA was amplified by RT-PCR using specific primers (sense, 5`-taa aag ctt atg agc tcc cgc atc gcc ag-3` and antisense, 5`-ccc ctc gag tta gtc cct aaa ttt gtg aat gtc-3`) that were designed based on the Cyr61 gene (GenBank ID: CR536519.1) by Takara (Shiga, Japan). The gel-purified PCR products were digested with the restriction enzymes, HindIII and XhoI (Takara, Dalian, China), and cloned into the eukaryotic expression vector, pcDNA3.1 (Invitrogen, American). The inserted sequence was confirmed by DNA sequencing.

According to the design rule for RNAi (Laganà et al., 2015), a fragment of Cyr61 gene (5`-AACAUCAGUGCACAUGTAUUG-3`) was used as the target siRNA (Cyr61-siRNA). As control, the sequence of Cyr61 siRNA was rearranged at random (5`-CAAUACAUGUGCACUGAUGUU-3`) to yield the corresponding random-siRNA (ram-siRNA). siRNAs were synthesized by Sangon Biotech Corporation (Shanghai, China).

Transient transfection of MDA-MB-231 cells with siRNA oligos (100 pmol) and recombinant plasmid pcDNA3.1-Cyr61 (4 µg/well) was carried out using Lipofectamineeq \o\ac(○,R)3000 Transfection Reagent Protocol (life technologies), according to the manufacturer’s instructions. Both of nontargeting siRNA and empty pcDNA3.1 vector were served as negative controls, respectively. These cells were harvested 24 h after transfection and used for further analysis.

### Cell Adhesion Assay

The adhesion assay of MDA-MB-231 cells to the HPMECs was assessed according to the method described previously by this lab (Yu et al., 2015; Yu et al., 2016). Briefly, HPMECs were seeded in 24-well plates and grown to 90% confluence in the ECM medium. Then, TNF-α (final concentration: 10 ng/ml) was used to activate HPMECs for 4 h. Rhodamine 123-labled MDA-MB-231 cells were washed twice using PBS and resuspended by ECM medium with a dose of metapristone, and co-cultured with the HPMEC monlayers (1:8) in each well for 2 h. DMSO (0.1%) was used as the vehicle control. Then, nonadherent cells were removed by PBS and ten visual fields for each well were selected randomly and taken pictures using a fluorescence microscope (Zeiss, Germany). Mean inhibition of adhesion for 10 visual fields was calculated by using the equation: % of control adhesion = [the number of adhered cells in treated group/the number of adhered cells in the control group] × 100%.

### Wound Healing Assay

MDA-MB-231 cells were seeded in 24-well plates with 5×10^4^/ml cells in complete medium and were reached over 90% confluence. The scratch wound was generated by using a pipette tip, and the floating cells were removed through washing with PBS. Then the PBS was instead by L-15 with different concentrations of metapristone (0, 10, 50, 75 µM) and the wound healing was recorded by using a fluorescence microscope (Zeiss, Germany) at 0 and 24 h. DMSO (final concentration: 0.1%) as vehicle control was added after wounding. At indicated time points, motility was quantified by measuring the average extent of wound closure. Each sample was assayed in triplicate in three independent experiments.

### RNA Extraction and Real-Time PCR

MDA-MB-231 cells (6×10^5^) were seeded in complete L-15 medium in 6-well plates and incubated over 24 h. Then, the medium was renewed with L-15 in the presence of metapristone (0, 10, 50, 75 µM). After incubation of 24 h, total RNA was exacted with Trizol reagent (Invitrogen, American) according to the manufacturer’s protocol and 4 µg RNA was converted to cDNA using PrimeScript™ RT reagent kit (TaKaRa, Dalian, China). The housekeeping gene, β-actin served as the internal control. Each real-time PCR reaction contained SYBRR) Premx Ex Taq II (Tli RNaseH Plus), PCR Forward/Reverse Primer, cDNA solution and dH_2_O. All PCR reactions were performed in triplicate using the mean value being used to determine mRNA levels. Relative mRNA expression levels for each gene were analyzed using the 2^-ΔΔCt^ method and normalized to the endogenous reference gene β-actin.

The main primers were as follows:

β-actin:
F: 5`-TGGCACCCAGCACAATGAA-3`
R: 5`-CTAAGTCATAGTCCGCCTAGAAGCA-3`
integrin αv
F: 5`-TTGTAAGTTGGCAGATCTTCCTAAGTT-3`
R: 5`-GATGGGTAGTGGCTGCACATAG-3`
integrin β1:
F: 5`-AATTGTGGGTGGTGCACAAAT-3`
R: 5`-TGGAGGGCAACCCTTCTTTT-3`
Cyr61:
F: 5`-TCT CGT TGC TCA TGA AATT-3`
R: 5`-TAG AGT GGG TAC ATC AAA GCT TCAG-3`

### Western Blot Analysis

MDA-MB-231 cells cultured in the present of metapristone (0, 10, 50, 75 µM) were lysed by RIPA. Then, the lysates were supplemented with HALT protease and phosphatase inhibitor cocktail (Thermo Scientific). Antibodies used for western blot analysis include Cyr61, integrin α_v_, integrin β_1_, and β-actin. Immunodetection of electrophoresis-resolved proteins was accomplished using enhanced chemiluminescence based on standard protocols, and signals were quantified with a quantitative digital imaging system (Quantity One, Bio-Rad) based on at least five replicates.

### Co-Immunoprecipitation

The formation of the Cyr61/integrin α_v_β_1_ complex in MDA-MB-231 cells was analyzed by co-immunoprecipitation and western blot according to the manufacturer’s instructions. Briefly, cells were lysed with IP Lysis/Wash Buffer (0.025 M Tris, 0.15 M NaCl, 0.001 M EDTA, 1% NP-40, 5% glycerol; pH7.4, containing 10 mM protease inhibitors (PMSF). Cell lysates (500 µl/15 × 10^5^ cells) were incubated with mouse anti-Cyr61 monoclonal antibody binding to aminolink plus coupling resin in pierce spin columns. Purified mouse IgG (Beyotime Biotechnology) was used as the negative control. The pierce spin columns were washed four times, and the bound proteins were released by Elution Buffer for western blot analysis with rabbi anti-integrin α_v_ and rabbit anti-integrin β_1_.

### Immunofluorescence Microscopy

MDA-MB-231 cells (1.0×10^5^) grown on 35 mm cell culture dish (NEST, GBD-35-20) were rinsed three times using PBS, and fixed in 4% paraformaldehyde for 30 min. Cells were then washed three times with PBS, and permeabilized with 0.2% Triton x-100 for 10 min. After washed three times with PBS again, the cells were blocked with 10% BSA for 30 min, then were incubated with mouse anti-Cyr61 (1:100) and rabbit anti-integrin α_v_ (1:500)/anti-integrin β_1_ (1:250) for 1–2 h in room temperature, rewashed, and incubated with FITC-conjugated goat anti-mouse IgG and CY3-conjugated goat anti-rabbit for 1 h. Finally, the cells were washed, and examined using a Leica TCS SP8 Spectral Confocal System.

### *In* *Vivo* Tumor Xenograft Study

Four or six-week-old female BALB/C nude mice were purchased from the Shanghai Laboratory Animal Center (Shanghai, China) and maintained under clean conditions. Then they were divided into experimental group and control group randomly, eight animals per group. Cells (5 × 10^6^) were resuspended in 200 µl of PBS, and injected into the lateral tail veins of mice, which had been orally gavaged with vegetable oil in the present of a dose of metapristone, 0 mg/kg (control), 2.5 mg/kg and 50 mg/kg for 3–4 days, respectively. After 5–7 weeks gavage, the lungs were removed, washed with PBS and fixed in 10% neutral buffered formalin. The number of lung tumor nodules was counted by visual inspection using a magnifying glass, then were paraffin embedded and stained with hematoxylin and eosin (H&E). The further study was to test the expression of Cyr61 and integrin α_v_β_1_ in lung tissue using immunohistochemical analysis assay. All of the animal experiments were performed in accordance with animal protocol procedures, approved by the Institutional Animal Care and Use Committee of Fuzhou University.

### Statistical Analysis

Data are presented as the mean ± standard error of the mean (SEM) or means ± standard deviations (SD). Statistical analysis was performed using the student’s *t*-test and one-way analysis of variance. A P-value less than 0.05 was considered statistically significant.

### Results

#### The Cytotoxicity Effect of Metapristone on the Growth of MDA-MB-231 and HPMEC Cells.

**Figure 1:**
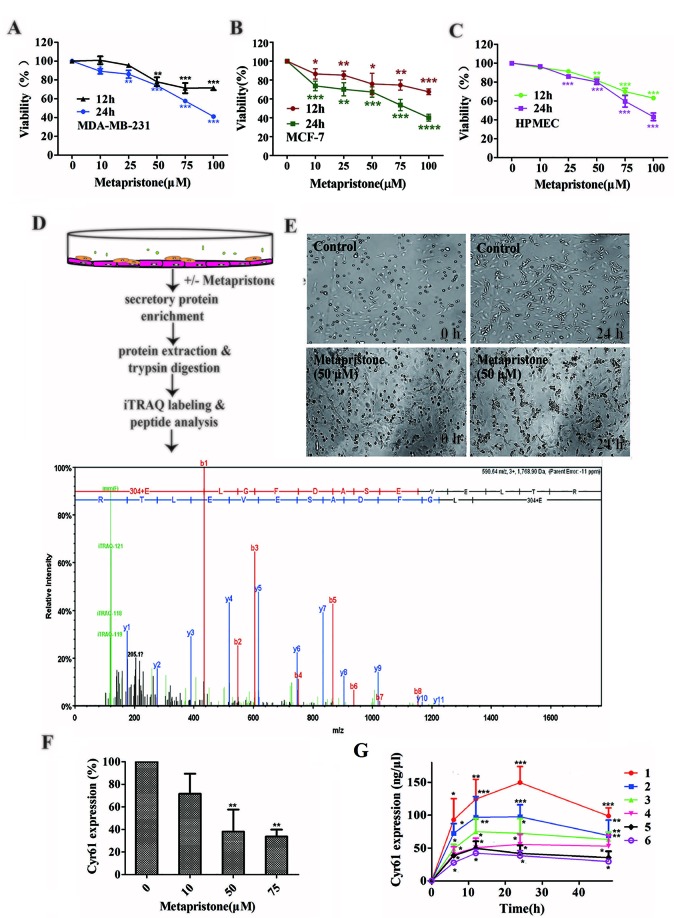
Secretion of Cyr61 from MDA-MB-231 and human pulmonary microvascular endothelial cells co-culture system and inhibition by metapristone of the secretion. **(A)** Effect of metapristone on MDA-MB-231 viability. **(B)** Effect of metapristone on MCF-7 viability. **(C)** Effect of metapristone on HPMEC viability. **(D)** Flow chart showing that MDA-MB-231 and HPMEC were co-cultured in the presence and absence of metapristone for 24 h followed by trypsin digestion and iTRAQ pharmacoproteomic analysis; The Cyr61 proteomic signal was shown at its corresponding m/z. **(E)** Confocal microscopy images showing the morphological changes by metapristone (50 µM) in the same spots where the two cell lines were co-cultured. **(F)** inhibition by metapristone of Cyr61 secretion. **(G)** time course of Cyr61 secretion (ELISA assay) under different conditions: 1) MDA-MB-231+ HPMEC; 2) MDA-MB-231+ HPMEC+ metapristone (50 µM); 3) MDA-MB-231; 4) MDA-MB-231+ metapristone (50 µM); 5) HPMEC; 6) HPMEC+ metapristone (50 µM). The data represent the mean± SEM (n = 3). *, *P* < 0.05, **, *P* < 0.01 and ***, *P* < 0.001 *vs.* the untreated control.

We evaluated the effect of various concentrations of metapristone on the viability of MDA-MB-231, MCF-7, and HPMEC cells for 12 and 24 h by MTT assay. As shown in **Figures 1A–C**, the viability of each group cells decreased in a dose- and time- dependent manner following metapristone treatment. Their IC_50_ values for metapristone (24 h) were 88.1 ± 3.2 (MDA-MB-231), 87.0 ± 2.7 (MCF-7), and 91.2± 2.5(HPMEC) µM, respectively.

#### Overview of Quantitative Proteomics and ELISA Validation

iTRAQ analysis was performed on the purified proteins extracted from the supernatant of co-culture system with or without metapristone (50 µM) treatment to find the metapristone-mediated anti-metastasis proteins. As represented by the flow chart in **Figure 1**. In total, 105 secreted proteins showed significant differences in metapristone-treated co-culture system (*P*-value < 0.05). Among these differentially expressed proteins (DEPs), 77 were up-regulated (**Table 1**) and 28 were down-regulated (**Table 2**). Pharmacoproteomic study showed that the expression of Cyr61 was signiﬁcantly down-regulated by metapristone by 60%, which was one of the most significantly changed proteins. To validate this, ELISA was performed. Compared with the control, the expression level of Cyr61 secreted from co-culture was down-regulated to 72%, 39%, and 36%, respectively, at 10, 50, and 75 µM of metapristone (**Figure 1F**), confirming that the proteomic data was reliable.

**Table 1:** Up-regulated proteins expressed in the conditioned media of MDA-MB-231 co-cultured with HPMECs.

No.	Score	%Cov	Accession number	Protein name	Peptides	Regulation (fold change)^a^
1	126	27.1	NP_003510.1	Cluster of Histone H2B type 1-L	7	1.59*
2	237	17.2	NP_001274523.1	Chloride intracellular channel protein 1	5	2.33*
3	218	26.1	NP_003290.1	Endoplasmin	5	1.77*
4	106	20.8	AAB59514.1	Apolipoprotein A-I	6	3.51*
5	552	29.8	NP_001596.2	Alanine–tRNA ligase, cytoplasmic	9	1.69*
6	267	30.0	NP_001245204.1	Sterile alpha motif domain-containing protein 3	5	3.15*
7	341	53.9	NP_001032752.1	Elongation factor 1-beta	11	6.51**
8	441	60.7	NP_005057.1	Splicing factor, proline- and glutamine-rich	10	1.75*
9	261	56.2	AAA58420.1	Caldesmon	5	7.50*
10	403	36.7	NP_004699.1	Programmed cell death protein 5	7	2.33*
11	230	51.4	NP_945189.1	Protein-glutamine gamma-glutamyltransferase 2	7	2.11*
12	137	24.2	NP_005491.1	SUMO-activating enzyme subunit 1	6	11.50**
13	1135	34.9	NP_001191020.1	40S ribosomal protein S10	5	3.55**
14	1123	43.9	NP_006127.1	F-actin-capping protein subunit alpha-2	10	5.24**
15	651	35.4	NP_060705.2	Cytosolic non-specific dipeptidase	7	7.21**
16	434	22.8	NP_057406.2	Ras-related protein Rab-14	7	10.15**
17	1019	58.2	NP_061819.2	Sialic acid synthase	9	1.85*
18	501	28.9	NP_001020092.1	60S ribosomal protein L9	5	3.26*
19	1101	33.9	NP_001304672.1	Elongation factor 1-delta	6	17.25**
20	348	29.0	NP_006005.2	EKC/KEOPS complex subunit LAGE3	6	1.87*
21	228	45.3	NP_001139699.1	40S ribosomal protein S20	9	6.56**
22	301	23.5	NP_001276978.1	General vesicular transport factor p115	8	2.76*
23	504	48.9	NP_001020.2	40S ribosomal protein S26	6	5.57**
24	216	24.1	NP_001306011.1	High mobility group protein HMG-I/HMG-Y	7	6.36**
25	259	22.4	NP_006704.3	Activated RNA polymerase II transcriptional coactivator p15	6	3.57**
26	133	45.6	NP_001339771.1	Suppression of tumorigenicity 18 protein	4	2.26*
27	1132	33.0	NP_001308414.1	40S ribosomal protein S19	11	3.69**
28	333	53.7	NP_002789.1	Proteasome subunit beta type-6	7	7.96**
29	309	44.2	NP_000966.2	60S ribosomal protein L11	5	4.23**
30	116	34.3	NP_001007.2	40S ribosomal protein S12	7	8.55**
31	243	35.1	NP_001034891.1	Cell division control protein 42 homolog	4	5.16**
32	634	44.3	NP_055277.1	U6 snRNA-associated Sm-like protein LSm1	10	6.33**
33	1005	30.7	CAA45860.1	Tetranectin	11	4.22**
34	1223	44.6	NP_996756.1	Serine/threonine-protein phosphatase PP1-alpha catalytic subunit	7	2.16*
35	164	17.1	NP_001186273.1	60S ribosomal protein L17	8	1.77*
36	422	20.6	NP_001254628.1	40S ribosomal protein S3a	11	2.89*
37	1117	52.3	NP_002892.1	Reticulocalbin-1	10	1.87*
38	311	22.1	NP_001135757.1	40S ribosomal protein S24	6	4.70**
39	118	28.3	BAB20429.1	Dihydropteridine reductase	5	11.33**
40	336	19.8	NP_006395.2	Endothelial protein C receptor	5	21.70**
41	559	43.7	NP_001001.2	40S ribosomal protein S6	7	9.18**
42	1224	29.4	NP_000963.1	60S ribosomal protein L7a	9	5.43*
43	406	23.4	NP_004517.2	DNA replication licensing factor MCM2	4	1.78*
44	518	24.5	NP_057215.3	Ras-related protein Rab-10	6	2.18*
45	217	33.8	NP_006560.3	Cell growth regulator with EF hand domain protein 1	5	3.54**
46	342	23.5	NP_001247436.1	40S ribosomal protein S3	6	1.90*
47	986	66.9	NP_072045.1	40S ribosomal protein S18	8	2.13*
48	1033	42.8	NP_006764.3	ATP-dependent RNA helicase DDX18	10	1.91*
49	557	67.9	NP_000509.1	Hemoglobin subunit beta	5	3.59**
50	1107	65.4	NP_001265308.1	ADP-ribosylation factor-like protein 6-interacting protein 4	6	3.22**
51	1091	21.6	NP_000508.1	Hemoglobin subunit alpha	6	11.90**
52	664	55.7	NP_789839.1	Proteasome activator complex subunit 3	5	13.27**
53	348	33.9	NP_001277255.1	Ubiquitin carboxyl-terminal hydrolase 38	7	5.93**
54	648	70.1	NP_001093163.1	60S ribosomal protein L31	8	2.77*
55	990	52.9	NP_001093256.1	Intercellular adhesion molecule 2	9	12.51**
56	115	19.5	NP_000345.2	Thyroxine-binding globulin	5	20.03**
57	667	33.5	NP_958845.1	Transcription elongation factor A protein 1	6	2.10*
58	1006	66.3	NP_001006.1	40S ribosomal protein S11	7	12.36**
59	852	44.4	NP_631908.1	Probable tRNA pseudouridine synthase 1	15	5.16**
60	223	66.1	NP_000306.1	Parathyroid hormone	9	2.53*
61	109	17.7	NP_001290555.1	60S ribosomal protein L10	11	6.26**
62	310	54.4	NP_000981.1	60S ribosomal protein L27a	8	11.15**
63	339	32.8	AAA51683.1	Alpha-2-HS-glycoprotein	9	2.23*
64	398	42.5	NP_808760.1	Histone H2A.J	7	2.57*
65	671	54.8	NP_001030168.1	60S ribosomal protein L14	12	9.31**
66	245	25.9	AAA02852.1	Aminoacylase-1	8	2.90*
67	279	45.9	NP_000959.2	60S ribosomal protein L4	6	12.14**
68	1013	56.2	NP_001022.1	40S ribosomal protein S28	5	7.42**
69	1102	63.6	NP_001230060.1	60S ribosomal protein L13	6	7.01**
70	686	73.0	NP_001307070.1	60S ribosomal protein L6	9	13.43**
71	354	55.4	NP_057018.1	Nucleolar protein 58	10	9.64**
72	1224	21.1	NP_001257907.1	IST1 homolog	10	2.66*
73	556	43.2	NP_001002.1	40S ribosomal protein S7	7	8.81**
74	352	26.1	NP_778224.1	Histone H4	4	2.90*
75	1039	79.4	NP_113584.3	E3 ubiquitin-protein ligase HUWE1	4	14.31**
76	601	29.6	NP_037377.1	Vacuolar protein sorting-associated protein 4A	7	16.37**
77	1096	58.4	NP_001307930.1	Translation initiation factor IF-2, mitochondrial	4	61.25**

a Regulations (fold-changes) of differentially expressed proteins in MDA-MB-231 cells (metapristone-treatment versus control). *P < 0.05; **P < 0.01.

**Table 2:** Down-regulated proteins expressed in the conditioned media of MDA-MB-231 co-cultured with HPMECs.

No.	Score	%Cov	Accession number	Protein name	Peptides	Regulation (fold change)^a^
1	699	23.3	P08779.4	Cluster of Keratin, type I cytoskeletal 16	11	0.47*
2	237	51.2	NP_001633.1	Amyloid-like protein 2	7	0.42*
3	1051	35.1	NP_000217.2	Keratin, type I cytoskeletal 9	7	0.51*
4	158	28.5	NP_004039.1	Beta-2-microglobulin	5	0.52*
5	435	17.3	NP_003245.1	Metalloproteinase inhibitor 1	5	0.35**
6	763	33.7	NP_004930.1	ATP-dependent RNA helicase DDX1	4	0.41**
7	501	19.1	NP_003013.1	SH3 domain-binding glutamic acid-rich-like protein	4	0.65*
8	722	61.4	NP_055635.3	Mitochondrial import receptor subunit TOM70	7	0.36**
9	1015	26.9	NP_001159506.1	Suprabasin	5	0.61*
10	238	47.2	NP_001339702.1	Junction plakoglobin	6	0.44**
11	122	23.4	NP_059118.2	Calmodulin-like protein 5	14	0.49*
12	633	61.1	NP_060275.2	Ras-interacting protein 1	6	0.57*
13	814	36.9	NP_003109.1	SPARC	16	0.40**
14	1226	19.7	NP_055463.1	E3 ubiquitin-protein ligase DZIP3	5	0.63*
15	1104	20.3	NP_005520.4	Basement membrane-specific heparan sulfate proteoglycan core protein	8	0.54*
16	439	21.7	NP_000349.1	Transforming growth factor-beta-induced protein ig-h3	8	0.59*
17	1615	36.3	NP_009016.1	Follistatin-related protein 1	4	0.63*
18	1532	34.1	NP_001545.2	Protein CYR61	8	0.39**
19	716	27.5	NP_001308350.1	Rho GDP-dissociation inhibitor 2	6	0.49**
20	374	34.5	NP_116020.1	Hepatoma-derived growth factor-related protein 2	10	0.56*
21	715	46.9	NP_000511.2	Beta-hexosaminidase subunit alpha	4	0.45**
22	1227	52.3	NP_002895.3	Negative elongation factor E	9	0.57*
23	1032	36.3	NP_079472.1	GrpE protein homolog 1, mitochondrial	7	0.61*
24	760	35.1	NP_002169.1	Insulin-like growth factor-binding protein 6	7	0.52*
25	1069	37.3	NP_001340245.1	Protein FAM49B	8	0.47**
26	419	29.1	NP_115729.1	Vacuolar protein-sorting-associated protein 25	12	0.56*
27	512	36.6	NP_004809.2	Probable ATP-dependent RNA helicase DDX23	6	0.53*
28	405	23.9	NP_001136155.1	Coiled-coil domain-containing protein 121	5	0.60*

a Regulations (fold-changes) of differentially expressed proteins in MDA-MB-231 cells (metapristone-treatment versus control). *P < 0.05; **P < 0.01.

### The Origins of Cyr61 Secreted

To explore whether MDA-MB-231/HPMEC co-culture increases Cyr61 secretion, we tested the concentrations of Cyr61 in different groups at 0, 6, 12, 24, and 48 h by ELISA. As shown in **Figure 1G**, the secretion of Cyr61 increased at the beginning, then saturated or decreased progressively. Cyr61 expression in co-culture group was higher than that in MDA-MB-231 or HPMEC monocultures. Both MDA-MB-231 cells and HPMECs could secrete Cyr61, but the former secreted more. The secretions of Cyr61 in metapristone groups were lower than that in non-treatment groups. These results indicated that the co-culture system provided a better microenvironment for expression and secretion of Cyr61 compared with monoculture.

To further investigate the relationship between metapristone and co-culture model, a time-lapse photography was taken, which showed that MDA-MB-231 cells suspended in culture tended to adhere to HPMECs on the dish, but this tendency was interfered by metapristone. The co-culture system in the presence and absence of metapristone showed almost 0% adhesion. At 24 h after co-culture, the control group reached 45% adhesion, whereas the metapristone-treated group reached only 20% adhesion (**Figure 1E**).

### Metapristone Inhibits Cyr61-Mediated Cell Migration and Adhesion

**Figure 2:**
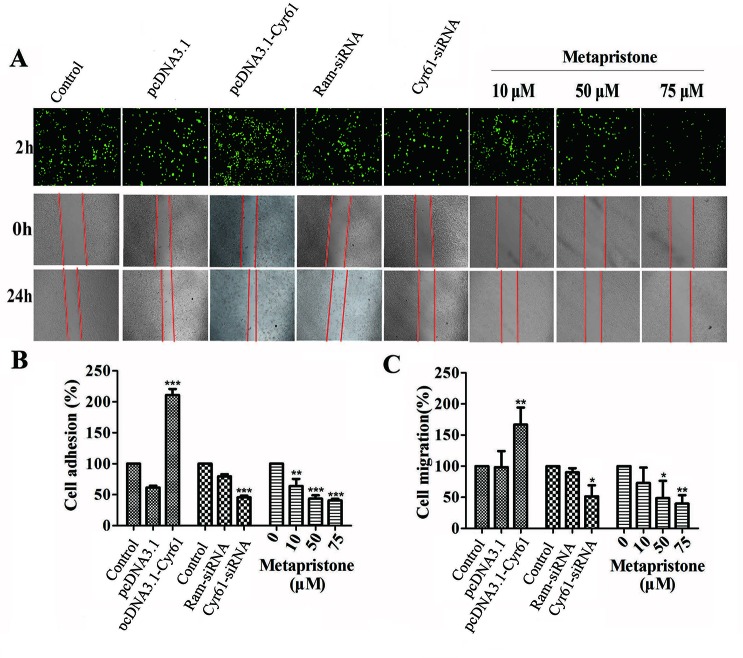
Cyr61-related cell adhesion and migration were mediated by metapristone. MDA-MB-231 cells were transfected with pcDNA3.1, pcDNA3.1-Cyr61 recombinant plasmid, or Ram-siRNA, Cyr61-siRNA for 36–48 h. **(A)** Upper panel: inhibition by metapristone of MDA-MB-231 cell adhesion to human pulmonary microvascular endothelial cells; note, the adhesion between pcDNA3.1-Cyr61 cancer cells and HPMECs was significantly enhanced. Whereas, the adhesion between Cyr-61-siRNA cancer cells and HPMECs was reduced in comparison with their controls. Metapristone caused concentration-dependent inhibition of the adhesion. Middle and lower panels: changes by metapristone in cell migration rate following the scratch assay. **(B)** Quantitative analysis of the adhesion between Cyr61-transfected cancer cells and HPMECs. **(C)** The MDA-MB-231 cells were transfected and treated differently, and the migration rate following the scratch assay was quantitatively determined; The data represent mean± SEM (n = 3), *, *P* < 0.05; **, *P* < 0.01; and ***, *P* < 0.001 *vs*. the controls.

First, we constructed the “pcDNA3.1-Cyr61” plasmid and synthesized the “Cyr61-siRNA” (see **Supplementary Information**, **Figure S1** and **S2**) to test the function of Cyr61 in metapristone-related anti-metastasis mechanics. Then, would healing assay was used to examine the effects of endogenous Cyr61 protein and metapristone on MDA-MB-231 cell motility. As shown in **Figure 2A**, the overexpression of Cyr61 enhanced cellular migration (170%) and the down-regulation of Cyr61 expression inhibited cellular migration (53%). MDA-MB-231 cells migration was inhibited by metapristone in a concentration-dependent manner by 74% (10 µM), 52% (50 µM), and 45% (75 µM), respectively (**Figure 2**). These results suggested that metapristone significantly inhibited Cyr61-mediated cell motility and wound closure at concentrations lowering than its IC_50_.

It is well known that tumor cells adhesion to the ECM is a fundamental step in cancer metastasis (Gilkes et al., 2014; Conlon and Murray, 2019). The adhesion of MDA-MB-231 cells to HPMECs was examined to determine whether metapristone can regulate cell adhesion at a non-cytotoxic concentration by Cyr61 pathway. Compared with the control, the adhesion rate of MDA-MB-231 cells was 218%, 48%, 63%, 44%, and 41%, respectively, for transfection groups (pcDNA3.1-Cyr61, Cyr61-siRNA) and metapristone treatment groups (10, 50 and 75 µM) (**Figures 2A, B**). Metapristone markedly inhibited the adhesion of MDA-MB-231 cells to endothelial monolayers in a concentration-dependent manner through the Cyr61-dependent mechanism.

### Metapristone Inhibits Adhesion and Migration of MDA-MB-231 Cells *via the* Cyr61/Integrin α_v_β_1_ Signaling Pathway

**Figure 3:**
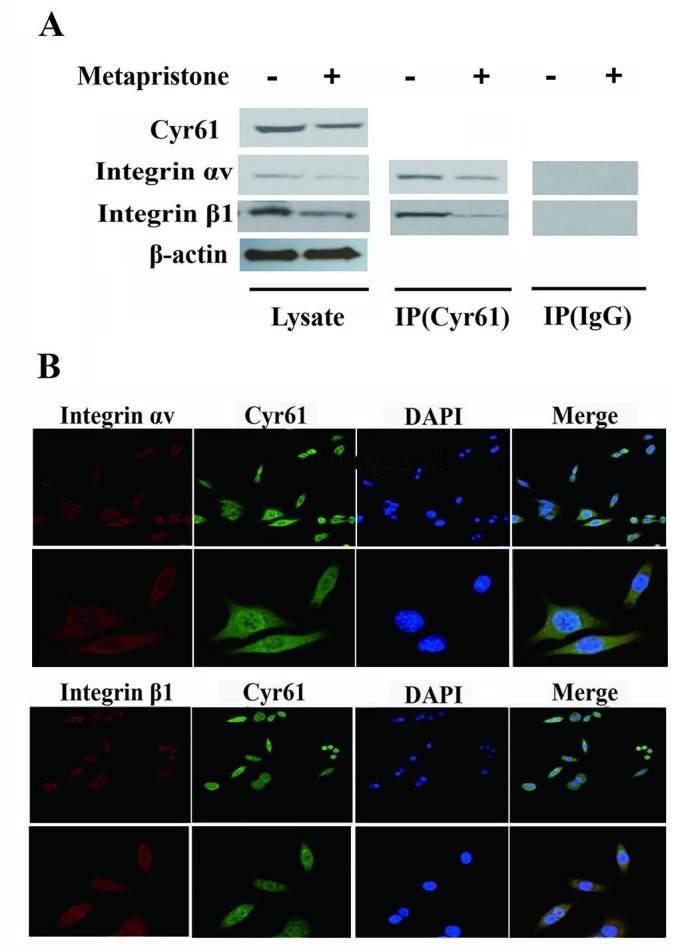
Formation of Cyr61/integrin αvβ1 complex in MDA-MB-231 cells. **(A)** Co-immunoprecipitation evidence: MDA-MB-231 cell lysates were added to aminolink-coupling resin columns that were individually coupled with anti-Cyr61 antibody (land 1 and 3), anti-integrin αv antibody (land 2), or anti-integrin β1 antibody (land 4). The bound complexes were eluted with the washing buffer and tested by western blotting with anti-integrin αv antibody (land 1), anti-Cyr61 antibody (land 2 and 4), or anti-integrin β1 antibody, individually. Samples of Lysis (before column; left), IgG, and Target (column elutes; right) after western blotting showed that the anti-Cyr61 antibody elute was integrin αv positive (land 1), integrin β1 positive (land 3), and Cyr61 positive (land 2 and 4). **(B)** Cyr61/integrin αvβ1 co-localization evidence: MDA-MB-231 cells were immunofluorescent-stained with Cyr61 (green), integrin αv (the upper two panels), or integrin β1 (the lower two panels; both in dark red) and DAPI (in blue for nuclear) dyes followed by confocal microscopic examination at amplification×20 (top panel) or ×40 (bottom panel). The merged images showed co-localization of Cyr61 with integrin αv and β1.

Previous reports have indicated that the CCN family interacts with integrin receptors to modulate cell biological functions (Holbourn et al., 2008). Therefore, we sought to identify the cellular adhesion receptor(s) through which Cyr61 may function. By using co-immunoprecipitation assay, as shown in **Figure 3**, integrin α_v_β_1_, as a new receptor for Cyr61 in the MDA-MB-231/HPMEC co-cultures was identified by immunoprecipitation combined with western blot analysis. We found that MDA-MB-231/HPMEC co-cultures promoted the formation of Cyr61/integrin α_v_β_1_ complex, which was inhibited by metapristone significantly. The results were further confirmed by confocal microscopy co-localized methods using immunofluorescent staining of the CYR1, integrin α_v_, and integrin β_1_ proteins in MDA-MB-231 cells (**Figure 3B**).

**Figure 4:**
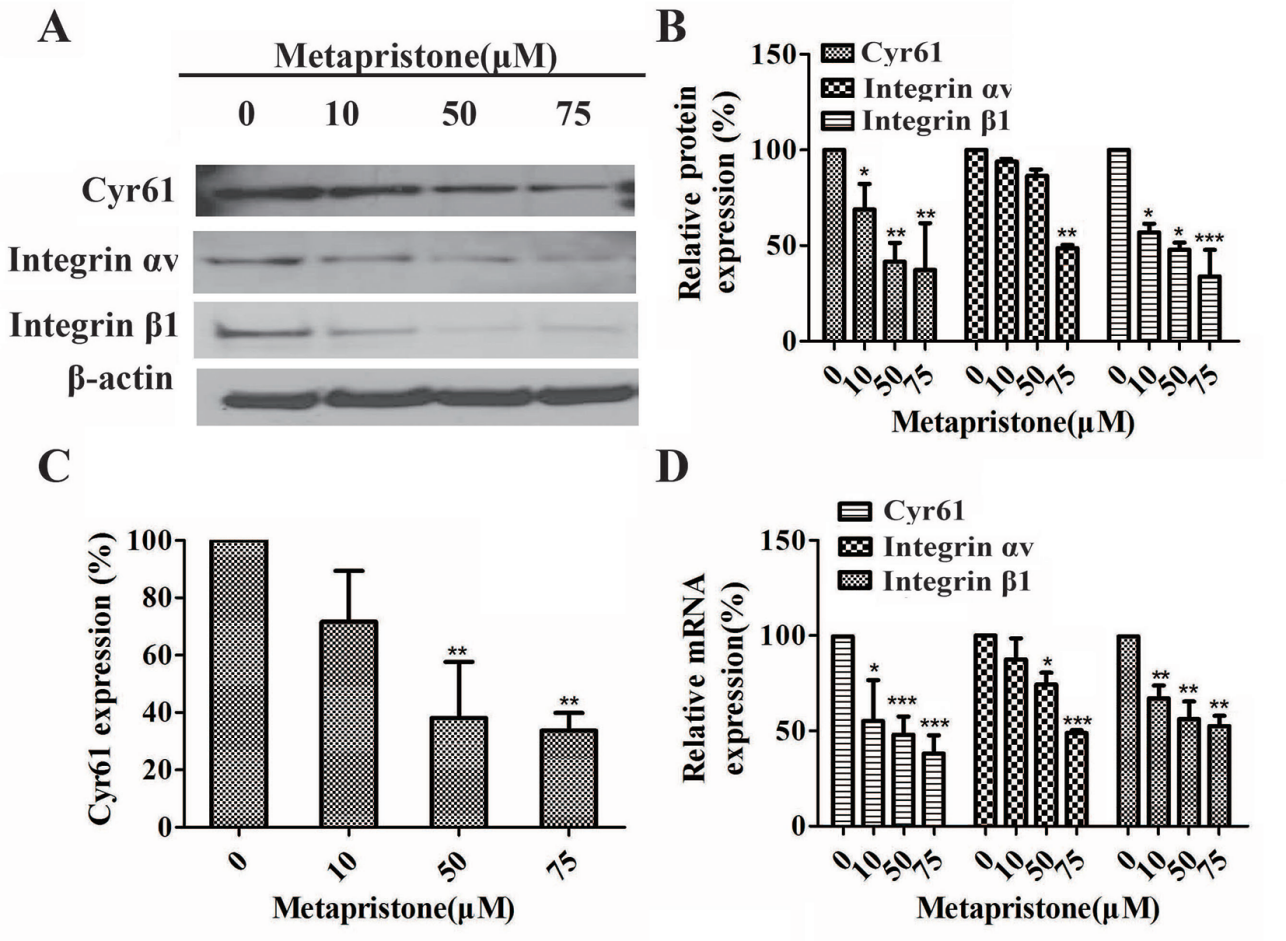
Inhibition by metapristone of expressions of Cyr61 and integrin αvβ1 in MDA-MB-231 cells. Western blot images **(A)** and the related quantitative analysis, **(B)** and quantitative ELISA analysis **(C)** of inhibition by metapristone of expressions of Cyr61 and integrin αvβ1 in MDA-MB-231 cells. **(D)** mRNA expression of Cyr61, integrin αv and β1 was inhibited by metapristone in a concentration-dependent manner. The data are expressed as the mean± SEM (n = 3). *, *P* < 0.05; **, *P* < 0.01, and ***, *P* < 0.001 *vs*. the untreated controls.

Furthermore, the effects of metapristone on the expressions of Cyr61, integrin α_v_, and integrin β_1_ on the protein and mRNA levels were determined using qRT-PCR and western blot analysis. The results showed that when the MDA-MB-231 cells were treated with different doses of metapristone, the expressions of Cyr61 and integrin α_v_β_1_ were dose-dependently decreased (**Figures 4A, B, D**). As the secreted protein, the expression of Cyr61 in cell culture was detected by ELISA assay. As shown in **Figure 4**, the significantly reduced expression of the Cyr61 was observed in the presence of metapristone. The metapristone might regulate cell adhesion and migration through Cyr61/integrin α_v_β_1_ pathway in MDA-MB-231 cells.

### Effect of Metapristone on Experimental Lung Metastasis *in* *Vivo*

**Figure 5:**
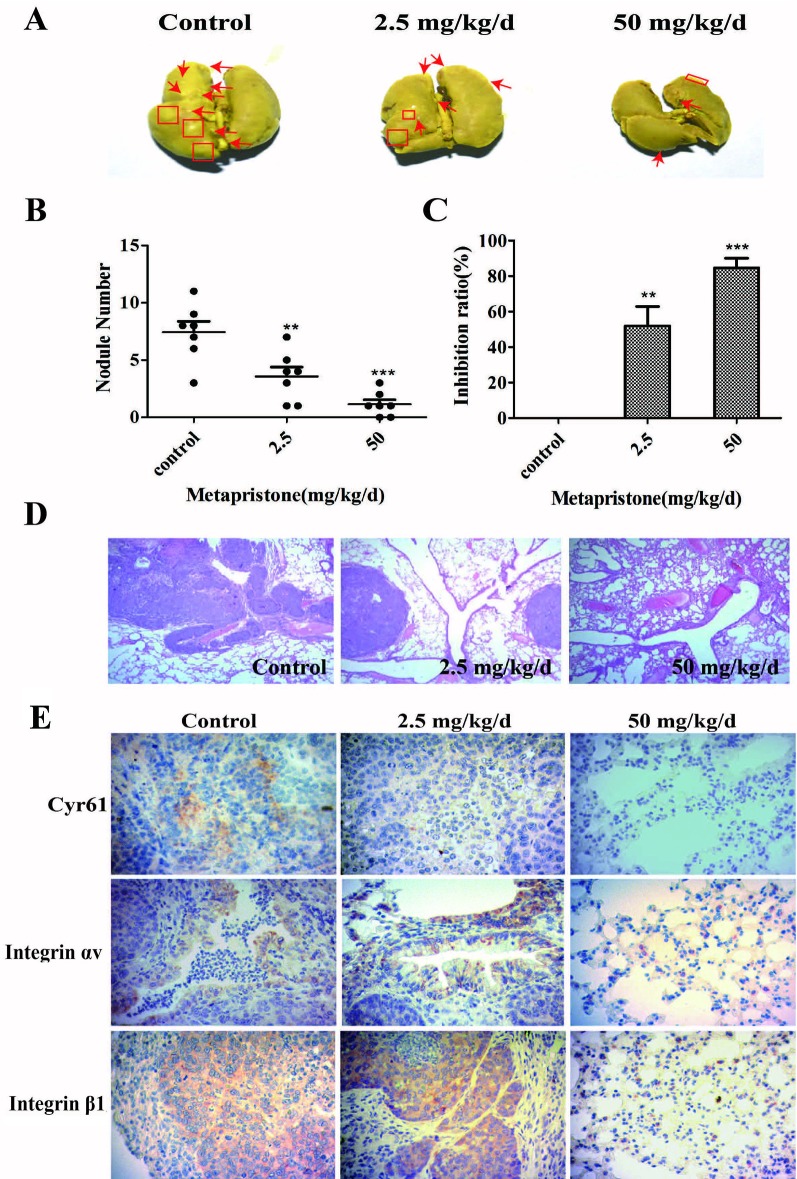
Metapristone inhibits lung metastasis of MDA-MB-231 cells *via* decreasing levels of Cyr61 and integrin αvβ1. **(A)** photography of mouse lung metastasis after six weeks of MDA-MB-231 inoculation *via* tail vein injection. The mice were pretreated with oral metapristone for three days before the inoculation followed by 6-week oral administration of metapristone. Control, drug vehicle; **(B)** quantitative comparison in mouse lung tumor nodules between the control and metapristone groups (n = 5/group); **P < 0.01, ***P < 0.001. **(C)** inhibition rate of mouse lung tumor metastasis after metapristone treatment; **(D)** hematoxylin–eosin staining of the lungs (amplification× 5); the arrows indicate metastatic foci that were significantly reduced by metapristone; **(E)** lung immunostaining with antibodies against Cyr61, integrin αv and integrin β1; the staining showed reduction in Cyr61/integrin avβ1 formation by metapristone.

The lungs are a frequent target of metastatic breast cancer cells (Landemaine et al., 2008; Oskarsson et al., 2011). Therefore, we further examined the anti-metastatic efficacy of metapristone on MDA-MB-231 cells using a xenograft mice model. **Figure 5** showed representative images of pulmonary metastases of MDA-MB-231 breast cancer in each group. We obviously observed that pulmonary metastatic nodules and the rate of lung tumor-metastasis from the mice treated with the metapristone were less than those of mice in control group (**Figures 5B, C**). In addition, the histological examinations (hematoxylin-eosin staining of various lung sections) showed the metastatic nodules colonized in the lungs of metapristone treatment groups were smaller than that of non-treated group with lower density (**Figure 5**). Furthermore, there was obvious effect on the expressions of Cyr61 and integrin α_v_β_1_ in these lung tissues (**Figure 5**) at different dose levels. By comparison, the positive areas of Cyr61 and integrin α_v_β_1_ were decreased with high dose metapristone.

## Discussion

Tumor microenvironment plays an important role in directly stimulating malignant cell metastasis. For example, normal endothelial and tumor cells usually communicate indirectly before or directly after adhesion, through a complex network of interactions to drive cellular differentiation, tissue structures formation, cancer invasion, and metastasis. One function of this communication is to exchange both soluble and insoluble signaling molecules, such as secreted proteins, miRNAs and exosomes. However, the underlying mechanism is not well understood.

In this study, we used a MDA-MB-231/HPMEC co-culture model to investigate the secreted proteins involved in cell adhesion/invasion, the crucial procedures of cancer early metastasis (Devis et al., 2017). iTRAQ technology exhibited superb performance in the quantitative proteomic study (Astier, 2010). Using iTRAQ-based proteomic approach, we identified 105 secreted proteins, showing significant differences in metapristone (a potential cancer metastasis chemopreventives)-treated co-culture secretome compared to the control group (*P*-value < 0.05) (**Figure 1** and **Table 1**). In particular, we found that MDA-MB-231/HPMEC co-cultures promoted the secretion levels of Cyr61 relative to MDA-MB-231 or HPMEC monocultures. In contrast, metapristone not only inhibited the Cyr61 secretion, but also prevented adhesion of MDA-MB-231 cells to HPMECs in morphology (**Figure 1** and **Figure 2**).

Cyr61 (CCN1), as the first cloned member of cysteine-rich protein (CCN) family, is a secreted, cysteine-rich, heparin binding extracellular matrix-associated protein (Planque and Perbal, 2003; D’Antonio et al., 2010). To date, a number of reports describe that Cyr61 is involved in many cell biological functions. For example, Cyr61 has been identified to mediate cell adhesion, migration, proliferation, apoptosis, and angiogenesis. Cyr61 is highly expressed in breast cancer (Sánchez-Bailón et al., 2015; Mayer et al., 2017; Yang et al., 2018), and is without a doubt associated with expression stage, tumor size, positive lymph nodes and age (Xie et al., 2001). The analysis indicated that blocking Cyr61 might be a potent method for TNBC breast cancer treatment. We previously reported how metapristone inhibited the adhesion and migration of MDA-MB-231 breast cancer cells through EMT-related pathway (Yu et al., 2016). However, the other mechanism remains largely unknown. In the present study, the effect of metapristone induction on Cyr61 activity to interfere cell adhesion and migration was examined by the transfection of siRNA-Cyr61/pcDNA3.1-Cyr61 into MDA-MB-231 (see **Supplementary Information**). Our results showed that overexpression/knockdown of Cyr61 significantly increase/decrease adhesion and migration of MDA-MB-231 cells, respectively (**Figure 2**).

A number of the activities of Cyr61 can be attributed to its interaction with integrin receptors (Kireeva, 1998; Jedsadayanmata et al., 1999; Chen et al., 2000; Grzeszkiewicz et al., 2001; Schober et al., 2002). For example, primary human skin fibroblasts adhesion to Cyr61 is dependent on integrin α_6_β_1_ (Leu et al., 2003), and activation-dependent adhesion of blood platelets to Cyr61 is mediated through interaction with integrin α_2_β_3_ (Jedsadayanmata et al., 1999). To understand Cyr61’s action in MDA-MB-231/HPMEC co-cultures, we sought to identify the cellular adhesion receptor(s) through which Cyr61 may function. We provided the first demonstration of the identification integrin α_v_β_1_ as a novel receptor for Cyr61 in MDA-MB-231/HPMEC co-cultures by immunoprecipation combined with western blot analysis (**Figure 3**). The expressions of Cyr61, integrin α_v_, and integrin β_1_ on the protein and mRNA levels were all down-regulated by metapristone in a dose-dependent manner. Furthermore, metapristone inhibited the formation of Cyr61/integrin α_v_β_1_ complex, which are correlated with cell adhesion and migration (**Figure 4**). Moreover, the circulating MDA-MB-231, developing lung metastasis in mice, could be effectively prevented by oral metapristone without significant toxicity ( **Figure 5**). Also, our studies demonstrated the obvious inhibition effect on the expressions of Cyr61 and integrin α_v_β_1_ in lung tissues after metapristone treatment.

**Figure 6:**
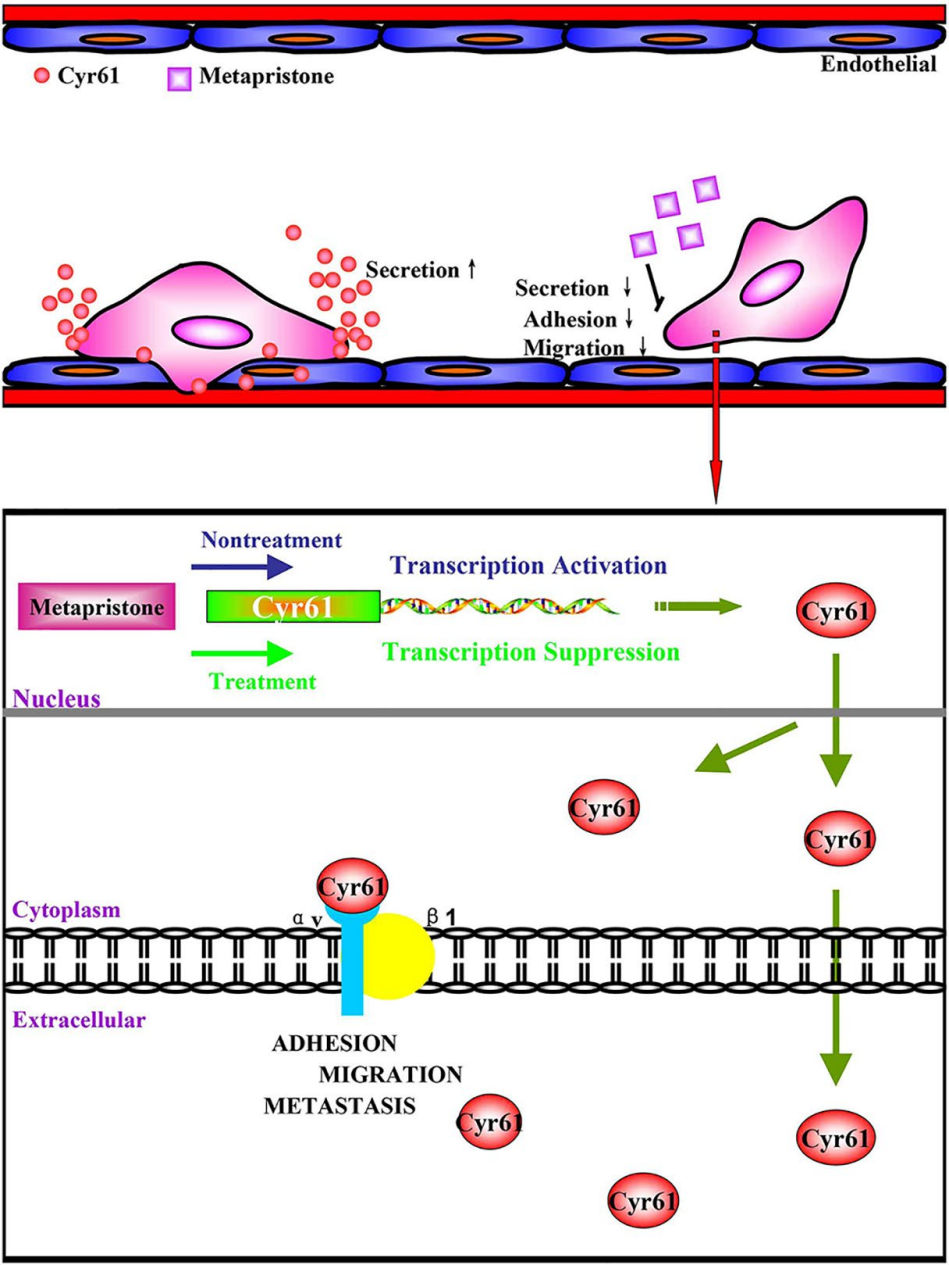
**(A)** possible mechanism of Cyr61 secretion and its inhibition by metapristone. Activated CTCs adhere to endothelial cells in the metastatic microenvironment, and the adhesion induces hetero-cellular communication and the resultant secretion of Cyr61 from CTCs, which forms Cyr61/integrin avβ1 complex to advance the adhesion/invasion metastasis. Metapristone inhibits the Cyr61 secretion, and the related adhesion/invasion process; **(B)** quantitative comparison in mouse lung tumor nodules between the control and metapristone groups (n = 5/group); **(C)** inhibition rate of mouse lung tumor metastasis after metapristone treatment; **(D)** hematoxylin–eosin staining of the lungs (amplification× 5); the arrows indicate metastatic foci that were significantly reduced by metapristone; **(E)** lung immunostaining with antibodies against Cyr61, integrin αv and integrin β1; the staining showed reduction in Cyr61/integrin avβ1 formation by metapristone.

Taken together, we have demonstrated for the first time that co-incubation of triple-negative breast cancer cell line MDA-MB-231 with HPMEC promotes the secretion of Cyr61 (CCN1), primarily from MDA-MB-231, which forms Cyr61/integrin α_v_β_1_ complex. Moreover, our data show that metapristone, a new chemopreventive, has the ability to inhibit TNBC cells adhesion and migration through down-regulation of Cyr61 and the formation of Cyr61/integrin α_v_β_1_ complex (**Figure 6**). Our data provide more details in understanding metastasis mechanism of microenvironment of tumor, especially under the tumor cells/endothelial cells co-culture condition, offer a cache of potential therapeutic targets, and more importantly, provide a molecular framework for clinical evaluation of metapristone as a potential cancer metastatic chemopreventive agent.

## Data Availability Statement

All datasets generated for this study are included in the article/**Supplementary Material**.

## Ethics Statement

The animal study was reviewed and approved by The Experimental animal ethics committee, Fuzhou University.

## Author Contributions

LJ and SY conceived and designed the experiments. SY and CY performed the pharmacoproteomic analysis. WW, SH, ML, JL, and YL carried out the cell biology experiments. XY and JM performed the animal experiments. CY and WW acquired and drew the pictures. LJ and SY wrote the manuscript. All authors read and approved the final manuscript.

## Conflict of Interest

The authors declare that the research was conducted in the absence of any commercial or financial relationships that could be construed as a potential conflict of interest.
