# Supplementary material for: RU486 Metabolite Inhibits CCN1/Cyr61 Secretion by MDA-MB-231-Endothelial Adhesion
Source: Front Pharmacol. 2019 Nov 20;10:1296. doi: 10.3389/fphar.2019.01296 (PMC6880622; doi:10.3389/fphar.2019.01296)
Supplement: Supplementary file 1 [file DataSheet_1.doc]

**Supplementary information**

**RU486 metabolite inhibits CCN1/CYR61 secretion by MDA-MB-231-endothelial adhesion**

Suhong Yu ^1#^, CuicuiYan^1#^, Wenjing Wu^1^, Sudan He^1^, Ming Liu^1^, Jian Liu^1^, Xingtian Yang^1^, Ji Ma^1^, Yusheng Lu^2^,  Lee Jia^1-2^*

1Cancer Metastasis Alert and Prevention Center, and Pharmaceutical Photocatalysis of State Key Laboratory of Photocatalysis on Energy and Environment, College of Chemistry; Fujian Provincial Key Laboratory of Cancer Metastasis Chemoprevention and Chemotherapy, Fuzhou University, Fuzhou 350002, China.

2 Institute of Oceanography, Minjiang University, Fuzhou, Fujian 350108, China

#These authors contributed equally to this work.

*Correspondence should be addressed to Lee Jia, Sunlight Building, 6FL; Science Park, Xueyuan Road, University Town; Cancer Metastasis Alert and Prevention Center, Fuzhou University, Fuzhou, Fujian 350116, China. Phone: 086-591-2286-7183. Email: [cmapcjia1234@163.com](mailto:cmapcjia1234@163.com) or [2697270856@qq.com](mailto:2697270856@qq.com)

*Keywords*: MDA-MB-231/HPMEC co-cultures, metapristone, Cyr61, integrin αvβ1, metastasis chemoprevention

Supplementary Figures:


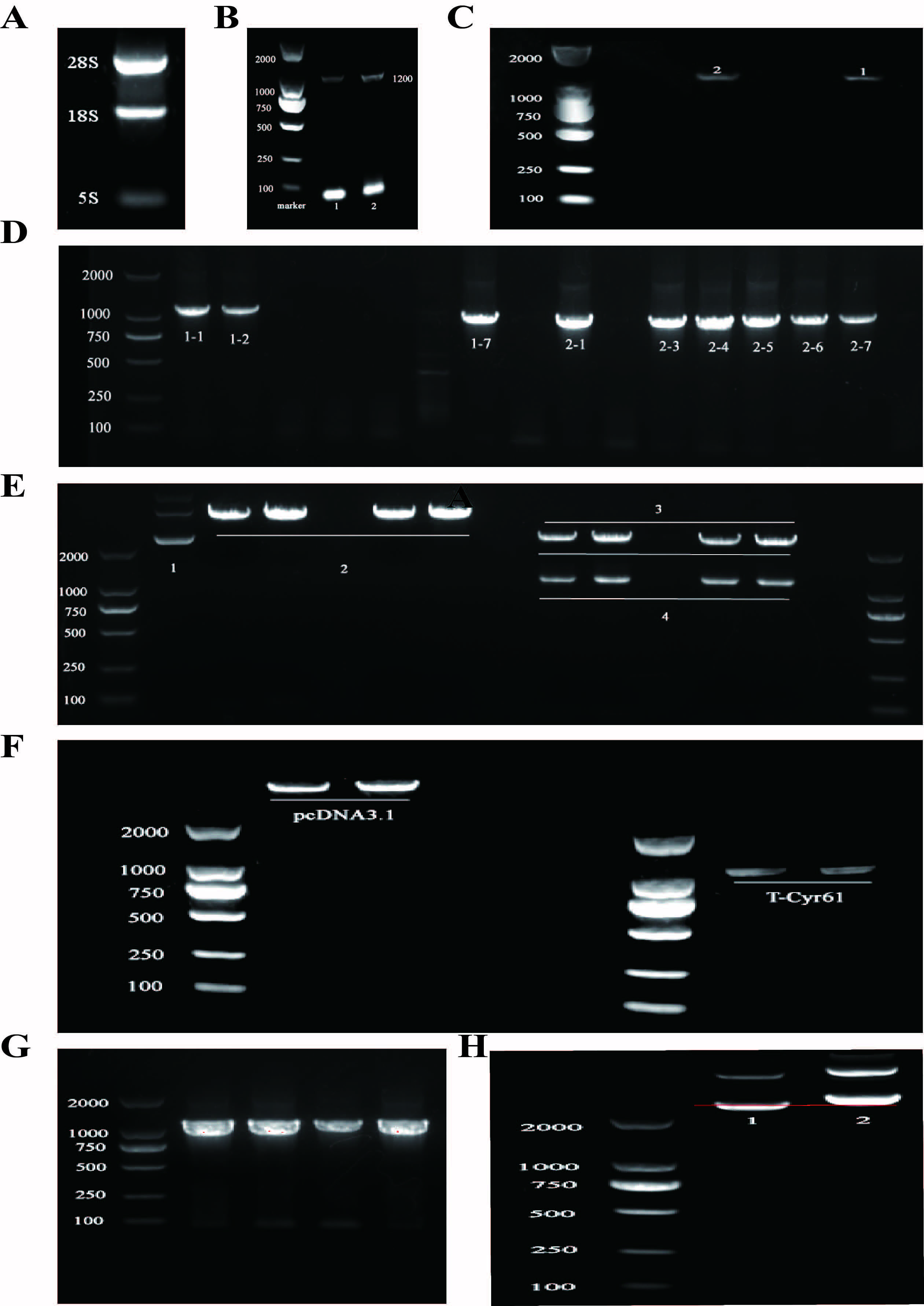


Figure-S1 The recombinant plasmid of pcDNA3.1-Cyr61. A: Purified RNA extracted from MDA-MB-231 cells. B,C,D: Full-length cDNA (Cyr61) synthesized from purified RNA and the suspected colonies were subjected to colony PCR with Cyr61 specific primers. E,F,G,H: The recombinant plasmid of pcDNA3.1-Cyr61 was testified by PCR and restriction enzymes (HindIII and XhoI) digestion analysis.


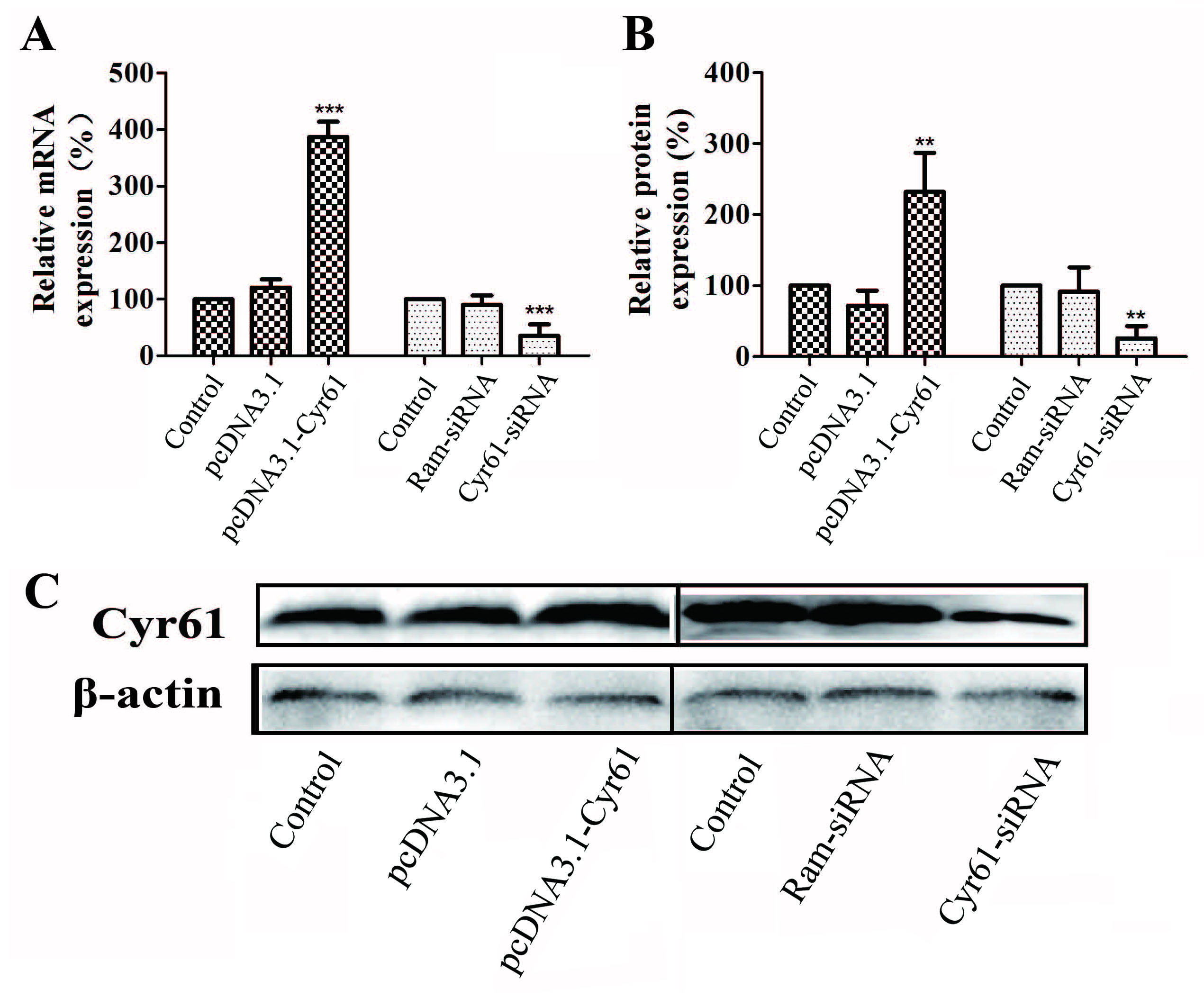


Figure-S2 Overexpression and knockdown of Cyr61 in MDA-MB-231 cells MDA-MB-231 cells were transfected with pcDNA3.1-Cyr61 recombinant plasmid and siRNACyr61 for 48 h, and the expressions of Cyr61-related mRNA (A) and protein (B, C) were determined. Control denotes the normal MDA-MB-231 cell line; pcDNA3.1, the same cell line transfected with pcDNA3.1; pcDNA3.1-Cyr61, the same cell line transfected with pcDNA3.1-Cyr61 to overexpress Cyr61; Ram-siRNA, the same cell line transfected with control siRNA; Cyr61-siRNA, the same cell line transfected with siRNA-Cyr61 to silence Cyr61 expression. The mRNA and protein expressions were measured by qRT-PCR and western blotting, respectively. The data are presented as mean± SEM (n=3). **, P< 0.01; ***, P< 0.001, compared with the controls.
